# Knowledge, attitude, and preferred strategies towards HIV/AIDS prevention among adolescents attending secondary schools in South Western Uganda

**DOI:** 10.4314/ahs.v21i3.14

**Published:** 2021-09

**Authors:** Esther Beebwa, Conrad Muzoora, Scholastic Ashaba, Sara Groves, Fortunate Atwine

**Affiliations:** 1 Department of Nursing, Mbarara University of Science and Technology, Mbarara, Uganda; 2 Department of Internal Medicine, Mbarara University of Science and Technology, Mbarara, Uganda; 3 Department of Psychiatry, Mbarara University of Science and Technology, Mbarara, Uganda; 4 Consultant, Massachusetts General Hospital Global Health, Boston; 5 Department of Nursing, Mbarara University of Science and Technology, Mbarara, Uganda

**Keywords:** Adolescents, attitudes, knowledge, HIV/AIDS, prevention

## Abstract

**Background:**

Globally, HIV/AIDS continues to rise among adolescents. Ugandan studies have examined knowledge and attitudes regarding HIV/AIDS among adult populations. This study specifically paid attention to this particular age group of adolescents 12–19 years.

**Aim:**

To explore HIV knowledge and attitudes among adolescents attending secondary schools Mbarara Uganda.

**Methods:**

A qualitative descriptive study was conducted in three secondary schools in South Western Uganda. Forty eight (48) adolescents with age range between 12–19 years were purposively recruited in the study. Data were collected from six focus groups and analyzed thematically. Ethical approval received from MUST (#05/10-17) and UNSCT (#SS4535) review committees.

**Results:**

Four themes emerged: Knowledge about HIV, sources of information, attitudes towards persons with HIV and prevention strategies. Most adolescents had the basic knowledge of HIV from multiple sources like social media, health workers, peers, and parents. Their attitudes toward individuals with HIV included compassion, shock, and uneasiness. Participants suggested prevention programs to be implemented in the schools emphasizing HIV education, life skills, sex education and the formation of peer groups.

**Conclusions:**

The findings showed that most participants had knowledge about HIV and how it can be prevented however few had knowledge gap thinking that HIV does not exist.

## Background

Human immunodeficiency virus (HIV) and acquired immunodeficiency syndrome (AIDS) remain one of the most serious global challenges the world is facing today and most especially in the sub-Saharan African countries^1^. Globally, approximately 590,000 adolescents between 15 and 24 years became infected with HIV in 2017 with 80% of these living in sub-Saharan Africa. A number of gaps exist for adolescent-specific HIV related data, however, eighty-two percent of the estimated 2.1 million adolescents aged 10–19 years living with HIV in 2012 were in sub-Saharan Africa, and the majority of these (58%) were females^3^

In Uganda, the prevalence of HIV among adolescents is higher in females (8.3%) compared to males (6.3%)^4^. Adolescents in Uganda contribute to 34.8% of the total population^5^ and are a significant population to target in HIV prevention. Information about adolescent prevalence in western Uganda is scanty. Nevertheless, HIV prevalence among women 15–24 was higher in urban than in rural sites ranging from.7–10 percent but with increased prevalence of 12.3 percent in the study area^6^. Adequate knowledge about HIV/AIDS is a powerful means of promoting positive attitudes and engaging in safe practices^7^ and a cornerstone in the fight against AIDS. Adolescents are especially vulnerable because they have limited or no historical knowledge of the beginning of the epidemic, limited current knowledge of the disease, poor attitudes toward HIV infected individuals, and little practice with prevention^8^.

Unsafe sexual behaviors place adolescents at higher risk of HIV infection. They are more likely to engage in the high risk behaviors of unsafe injection drug use, and exposure to contaminated blood products or unsterilized skin-piecing procedures^9^. Risk taking is part of normal adolescents' behavior whether it is for fun or thrill, to meet expectations of the peer group, or to try on the adult role^10^. It allows the adolescent to form their new identity, assess their new world, and experiment with how they make decisions^11^. Adolescence is clearly a time when young people start to explore their sexuality, a period when access to sexual health information is important^12^. It is also an important time for parents and other significant adults to help foster good decision-making regarding counseling and testing. Adolescents who remain untested they do not get counseling lessons which would help them not to engage in risky behaviors that can cause transmission of HIV^13^. Adolescents frequently are looking for a good listener, someone who is willing to try to understand their conflicts and help with making good choices^14^. Despite the need to help adolescents understand the risk of exposure to HIV, there are structural challenges of limited resource settings including social, economic, political, and environmental factors that limit access to relevant programs^15^.

Ugandan HIV prevention programs have included a combination of risk avoidance and risk reduction allowing unique success in reducing HIV prevalence^16^. These programs have included comprehensive education campaigns, safe blood transfusions and encouragement of safe sex by use of condoms, abstinence, and faithfulness^16^. There was a significant fall in the annual number of new infections between the late 1980s and mid-1990s, which led to a reduction in HIV prevalence. This however, is now changing, especially in the adolescent population, where the prevalence has been increasing^18^. This study explored the knowledge, attitudes and preferred strategies towards HIV/AIDS prevention among adolescents attending secondary schools in South Western Uganda. This information can contribute to national HIV prevention policies that appeal to adolescents through shared messages, better education and intervention in the schools to create a more effective prevention programs.

## Methods

### Study area Study design and study participants

This was an explorative qualitative study which used semi structured focus group guide with aim to provide an understanding of a topic about which little was known^19^. In this case focus group discussions were conducted among secondary school students in Mbarara Municipality Uganda. This area lies in South Western region; it is about 270 KM away from Kampala the main capital city of the country. The region is mainly inhabited by a majority of subsistence farmers. Mbarara municipality has 9 public secondary schools and 35 private schools. Three secondary schools were purposively selected, one for girls only, another for boys, and a mixed-gender school in order to balance gender.

In Uganda all schools whether public, private or faith based, follow guidelines, policies, regulations and standards from the Ministry of Education and Sports. The syllabi/curriculum is the same because they do the same examinations^20^. However in terms of discipline, students in faith based schools tend to be highly disciplined because they are influenced by religious beliefs^21^. The purpose of study participants recruitment was to provide rich information^22^ by using selected mixed schools from public, private and faith based.

The adolescents were assessed from an area of high prevalence of HIV in Uganda. The strength of Focus group discussion lies in the kind of interaction that emerges during the debate^19^.

### Data collection

To allow enough time, for successful group discussions one session was done each day. Two (2) FGDs were conducted for each school making six FGDs in total; Each group comprised of eight (8) to twelve (12) participants. Before focus group discussion, trained moderator and assistant were first introduced to students by the first author (principal investigator) together with the teacher who worked as a coordinator. The teacher later left the interview venue to give freedom and privacy to students. The first author (Principal investigator) gathered the selected students of ordinary level alone and advanced level alone. Brief explanation, of the aims and purpose of the study were given, to the students as they were also requested to participate in the study. The first author (Principal investigator) applied the skills acquired through series of practices before data collection. FGDs of every school consisted of students of different age groups 12–14, 15–17 and 18–19 years. The participants were encouraged to talk freely because they were familiar with each other since they were from same school and same class and most importantly they were of the same age group. The focus group discussions were let by experienced moderator guided by pretested open ended questions, also using probes and pauses in order to yield powerful information from all the participants^18^. Data was collected using a pretested semi-structured focus group guide and the discussion was digitally recorded. Each session lasted for 45–60 minutes in a neutral, agreeable, and convenient place without interruption. In order not to disrupt school routine activities and curriculum, time for FDGS was given by school administration.

The FGDs were conducted in English since it is the language of communication in the schools and all the students could communicate well. Information related to the social-demographic characteristics of the participants was collected first by assistant moderator assisted by the first author (principal investigator). Young assistant moderator was used to increase trust and disclosure among the students.

### Data analysis

All audio recordings were transcribed verbatim, Interviews were read numerous time and by the Principal investigator and research assistants to identify relevant themes in relation to the aim of the study. Data was analyzed manually in six phases: familiarization with the data, generating initial codes, searching for sub-categories among the codes, reviewing the merged sub-categories categories, defining and naming the categories, and then producing a final report.

### Trustworthiness

Measures to ensure credibility and dependability were established as described by^22^.

providing quotations from participants so that the categories can be judged. Data analysis triangulation was conducted to establish honesty in the data by the first author, FA, SG, the two double checked the content to confirm the relevance of categories as they experienced in qualitative data. CM verified the content because he has vast experience in HIV management as he is a physician. In order to minimize data collection biases, the research members were not part of the school members. In addition by providing reach description of each part of research process it would be possible to replicate the study to ensure transferability.

### Ethics approval and consent to participate

Ethical clearance was obtained from the Education Officer of Mbarara Municipality. Institutional ethics approval was granted by Research Ethics Committee of Mbarara University of Science and Technology (#05/10-17), Uganda National Council for Science and Technology (#SS4535) The firs author (Principal investigator) after getting clearance from all relevant committees went head to present permission letters from Education officer to all head teachers the secondary schools selected. The participants were informed about the purpose of the study prior to their recruitment and they consented/assented before data collection began. All participants above 18 years were provided with consent forms and those less than 18 had permission from their parent/guardian through the head teachers and then assents were obtained. Since there was a minimal foreseeable risk the consent from the head teachers was used for the minor students.The participants were assured of their rights either to participate or to withdraw from the study at any point and they did not need to answer questions that made them uncomfortable. Confidentiality was assured by not revealing their identities and no information was used that could identify an individual participant.

## Results and discussion

### Social demographic characteristics

We conducted a total of 6 Focus Group Discussions (FGDs) of 8 participants each (Total n=48) both males and females aged 12–19 years. Within each school, we ensured equal numbers for each level of education (n=8 for O-level and N=8 for A-level). The sex was purposively balanced (Females =24 and males =24). Because of this stratification, age ranges were equally balanced (n=24 for ages 12–15 and n=24 for ages 16–19)

Four themes emerged: Adolescent's knowledge about HIV, sources of information about HIV and attitudes towards persons with HIV and HIV prevention strategies.

### Knowledge regarding HIV

All respondents knew about HIV and most of them indicated that symptoms and signs do not show quickly and HIV is an incurable disease. Students, who were older^24^ from advanced classes of five and six, had better knowledge how HIV is transmitted. Others were knowledgeable about its treatment. This information can be supported by participants' responses as follows:

“*... ..people don't know that they have itHIV before they go for checking to know, it is always too late since the disease does not cure*” (GP1, P3).

“*It is an infection which is brought by blood &sexual contact and which can never heal (GP1, P1)”. “HIV means a disease, which can get everyone, the rich or poor, and it has no cure*” (GP1, P8).

HIV infection was perceived by adolescents to be a dangerous condition that does not discriminate one's status. Knowledge regarding HIV transmission seems to be adequate and it is reported that better knowledge leads to a later on set of sexuality among adolescents^23^. Despite a noteworthy HIV knowledge among the adolescents, there was also a smaller group of students who had considerable misinformation about HIV that it does not exist as manifested:

“*Me HIV/AIDS doesn't exist but it may be an allegation by the whites because they want to scare us*” (GP4, P9). This observation is not different from another study from Ugandan adolescents' report with misconception that HIV can be cured^4^

### HIV information source

The adolescents reported the main source of information on HIV/AID to be:

Health care providers, mass media, infected people, parents, peer groups and teachers. Other media included churches and schools. Below are some of the illuminations of the participants:

“*There is a program that was started by health teams called The Presidential Initiative on AIDS Strategy to Youth (PIASCY). They usually move in primary schools starting with primary five to primary seven.^7^). ....we used to get information from medical personnel who come from big hospitals; (regional referral hospitals) some come from clinics or health departments*” (GP1, P4).

“*Social media, Radios and televisions communicate about AIDS in the world. That's where we have been getting information*” (GP1, P5).

These students described clubs, songs, poems, and other educational activities, in their schools as a strategy to care for family and friends who had HIV. Many of these were through government programs that had strong peer components as: friends, straight talk package, and peers educational activities.

“*Some adolescents share information with their age mates... our friends share experiences*” (GP1, P6).

It is noted that health care providers in Uganda have played a big role to extend HIV care to schools. This is in contrast with other countries in Africa. In Ghana and South Sudan students get HIV information mainly by means of printed material and mass media using television means^21^, ^22^. However, students in Uganda like to use mass media and cell phones in order to make Centre calls for clarity^24^. This observation supports the idea that students in Uganda are not allowed to possess cell phones. This study highlights that there are limited educational programs for adolescents regarding HIV prevention strategies like elsewhere^25^.

Some of the participants were getting information on HIV/AIDS from peers of which was misleading. False information could be the reason for the increased HIV prevalence reported among the adolescents in the schools^4^.

Information from parents was reported to be very indirect and obscure in this study HIV/AIDS prevention discussions between parents and their children are still rare in Uganda due to the more conservative, religious and traditional beliefs of the Ugandan culture^24^.

### Attitude towards persons with HIV

This theme emerged from two subthemes: Attitude towards peers with HIV and the affected teachers.

### Attitudes towards peers with HIV

The respondents expressed a variety of feelings when asked how they would feel if a friend or roommate and a teacher had HIV/AIDS. Most of them felt compassion for the mentioned people especially those who were born infected. Some felt uncomfortable to be closer with them and preferred to observe social distancing. However, others felt sympathetic and reported that they would treat the HIV infected persons like a normal individual. The following statements reflect these attitudes.

“*The issue is, since I found my roommate having HIV/AIDS, what I have to do is, comfort him and guide him because nowadays things are rough when he finds out he is sick he hates himself*”(GP1, P7).

“*If I found out that my roommate has HIV/AIDS, honestly speaking, I would be scared*.” (GP5, P4).

### Attitudes towards teachers with HIV

Most participants reported that if their teachers had HIV they should retain their teaching positions since they can be exemplary.

“*Yes, because she knows the difficulties with it. She can counsel us about the consequences he/she has met and then to us, we can also be sure of information regarding difficulties and consequences that go with HIV*” infection (GP3, P5).

“*I think people with HIV can be good teachers because they are speaking from the experience*,” (GP4, P3).

With respect to attitudes, adolescents expressed very different feelings. These adolescents revealed common hidden negative attitudes. In a Malaysian study half, the adolescents were afraid to visit a friend with HIV even though they knew that HIV could not be transmitted with casual contact 25. These mixed responses were also found in other studies^26^. As part of a prevention program, stigma is still an important component, and in all the studies increased knowledge relieved some of the discrimination.

### HIV preferred prevention strategies

Participants reported sexual education on abstinence, avoiding watching pornographic films, use of condoms, as the main ways of HIV/AIDS prevention strategies.

“*----------i think schools should encourage sex education so that they can teach young children about HIV/AIDS and its effects and also to encourage children to abstain, stop watching pornographic films and use condoms, so that the remaining population can be safe*” (GP3, P2).

Students openly shared sexual experiences, being seduced, watching pornography, being free from parental control, and other adolescent behaviour.

Participants reported sexual education as one of the main HIV/AIDS prevention strategies. This implied that the schools needed to reinforce the sex education curriculum and to add more programmes. This was supported in the meta-analysis of school-based sex education programs in low and middle-income countries where these programs were found to be very effective^27^. This study revealed that more HIV information was provided by the health workers. The adolescents therefore, suggested that parents, teachers and sex educators should be more involved in HIV/AIDS education and prevention strategies. Other suggested strategies included provision of resources for HIV testing services to adolescents and avail condoms.

Abstinence from sexual intercourse and delayed initiation of sexual behavior were among the central aims of HIV prevention efforts for adolescents. Promoting positive attitudes towards HIV prevention, and adolescents taking responsibility for their own safety were important strategies^28^. Based on the focus group discussions in this study, it was clear that although abstinence was recommended it might not be practical. A program might include strategies, which combine both knowledge and behavioral skills, to promote risk-reduction behavior.

### Implications

This study identified adolescent friendly interventions aimed at preventing the spread of HIV among adolescent in South Western Uganda. This information can contribute to national HIV prevention policies that appeal to adolescents through shared messages, better education and intervention programs in the schools, with improved approaches by parents and healthcare professionals

## Conclusion

Most participants were knowledgeable about HIV because they knew how it is transmitted. However, some had little information and they thought HIV did not exist. This has made it difficult for some HIV prevention programs to succeed. More strategies are therefore needed like sensitization and attitude change. This is the reason HIV and sex education are needed in Ugandan secondary schools

## Figures and Tables

**Table 1:** Participant socio demographic characteristics

Variable	Number
**Sex**	
Male	24(50%)
Female	24(50%)
**Age**	
12–15	24(50%)
16–19	24(50%)
**Education Level**	
Ordinary	24(50%)
Advanced	24(50%)
